# Letting Babies Walk Too Early

**Published:** 1890-01

**Authors:** 


### LETTING BABIES WALK TOO EARLY.

The senseless conduct of many parents in encouraging their babies to walk is productive of lasting injury. Long before their soft bones ought to have any strain put upon them, you will see these poor infants encouraged to stand and even to walk, and by the time they are fourteen or sixteen months old, their little legs have been bent very considerably, and the greatest care is needed to straighten the bones again. Sometimes unsatisfactory operations are required, at other times cumbrous appliances have to be put on which cause the poor child much trouble, and represent a very considerable outlay.

Why not have a little patience? All in good time the tiny creature will learn to walk, and will walk well and safely, without danger of its tender bones bending. Under a year, let the child crawl, but do not let it walk, seldom indeed stand, and then only for a minute, and from one year to eighteen or twenty months, do not allow it to walk much; and when grown-up people help it to walk, they ought to stoop very considerably, and not put any strain on its feeble little body. Many a cripple owes its life-long misery to the injudicious encouragement of proud but foolish parents, who could not be induced to wait for nature's good time.

